# From Knowledge Transmission to Knowledge Construction: A Step towards Human-Like Active Learning

**DOI:** 10.3390/e22080906

**Published:** 2020-08-18

**Authors:** Ilona Kulikovskikh, Tomislav Lipic, Tomislav Šmuc

**Affiliations:** 1 Department of Information Systems and Technologies, Samara National Research University, Moskovskoe Shosse 34, 443086 Samara, Russia; 2 Division of Electronics, Ruđer Bošković Institute, Bijenička cesta 54, 10000 Zagreb, Croatia; tomislav.lipic@irb.hr (T.L.); tomislav.smuc@irb.hr (T.Š.)

**Keywords:** item information, pool-based sampling, multiple-choice testing, item response theory, active learning, deep learning

## Abstract

Machines usually employ a guess-and-check strategy to analyze data: they take the data, make a guess, check the answer, adjust it with regard to the correct one if necessary, and try again on a new data set. An active learning environment guarantees better performance while training on less, but carefully chosen, data which reduces the costs of both annotating and analyzing large data sets. This issue becomes even more critical for deep learning applications. Human-like active learning integrates a variety of strategies and instructional models chosen by a teacher to contribute to learners’ knowledge, while machine active learning strategies lack versatile tools for shifting the focus of instruction away from knowledge transmission to learners’ knowledge construction. We approach this gap by considering an active learning environment in an educational setting. We propose a new strategy that measures the information capacity of data using the information function from the four-parameter logistic item response theory (4PL IRT). We compared the proposed strategy with the most common active learning strategies—Least Confidence and Entropy Sampling. The results of computational experiments showed that the Information Capacity strategy shares similar behavior but provides a more flexible framework for building transparent knowledge models in deep learning.

## 1. Introduction

**Figure 1:**
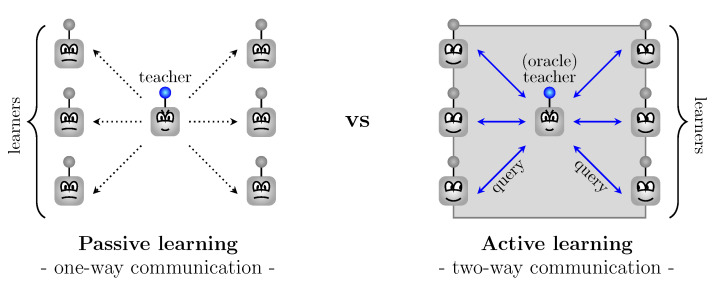
The concepts of learning environments: Passive vs. Active.

The passive learning technique normally requires an enormous amount of labeled data that has to provide the correct answers (see Figure 1). An active learning environment guarantees better performance while training on less, but carefully chosen, data which reduces the costs of both annotating and analyzing large data sets [1,2,3,4,5,6,7,8,9,10]. In uncertainty sampling, which has been reported to be successful in numerous scenarios and settings [11,12], a machine requests instances which cause uncertainty. This leads to the optimal leveraging of both new and existing data [13].

The process of querying the information imitates a classroom instructional method that actively engages learners in the learning process [14,15,16]. They replace or adapt their knowledge and understanding based on prior knowledge in response to learning opportunities provided by a teacher. This contrasts with a model of instruction whereby knowledge is transmitted from the teacher to learners, which typically presents passive learning. Active learning in an educational setting integrates a variety of strategies and instructional models chosen by a teacher to contribute to learners’ knowledge [17].

Hence, machine active learning strategies are still expected to be more versatile and self-sustaining. In particular, deep neural networks demonstrate remarkable performance on particular supervised learning tasks but are not good at telling when they are not sure while working in an active learning environment. The output from the softmax layer usually tends to be overconfident. Besides, deep neural networks have grown so complex that it seems practically impossible to follow their decision-making process [18].

In this study, we intend to inspect humans and machines reasoning processes [19,20,21,22,23] in order to understand how machines make predictions in an active learning environment. Rather than improving performance, we explored whether we can explain how machines come to decisions by imitating human-like reasoning in multiple-choice testing [24,25,26,27,28,29]. We suggest a new uncertainty sampling strategy based on the four-parameter logistic item response theory (4PL IRT) [24] we call Information Capacity. The strategy guarantees the performance similar to the most common uncertainty sampling techniques—Least Confidence and Entropy Sampling—but allows creating more transparent knowledge models in deep learning.

In deep neural networks, we have little visibility into the understanding of how models come to conclusions. This happens because we do not know how learning is supposed to work. While training a model, we iterate with better data, better configurations, better algorithms, and more computational power, although we have little knowledge why that model converges slowly and generalizes poorly. As a result, we do not have much control over rebuilding that model—it is not transparent [18,30,31].

Information Capacity brings with it a new interpretation of learning processes to enlighten “black-box” models. In contrast to Least Confidence and Entropy Sampling, the proposed strategy relies on neural network architectures to model learners’ behavior, where neurons or network weights of network classifiers are considered to be a group of learners with different proficiency in classifying learning items. Information Capacity ensures more flexible deep architectures with explainable and controllable learning behavior, not restricted to connectionist models.

### Related Work

- Deep active learning. Active learning of deep neural models has hardly been considered to date. The prominent related studies report minimizing test errors and computational efforts [32,33,34,35,36], taking some directions towards interpretability in deep learning [37]. This study approaches another major issue within the context of transparency—a lack of reasoning in deep neural models.
- IRT-based deep learning. Item response theory has been successfully used in solving machine and deep learning problems [38,39,40,41]. They mostly focus on improving generalization ability through optimizing the parameters of IRT models. Rather than optimizing hyperparameters  [42] via IRT model-fitting, we aimed to find meaningful interpretations of deep networks reasoning with learning behaviors.
- Meta active learning. The reported studies mostly focused on increasing the accuracy of classification with adaptive optimization schemes [1,5,43]. Instead, we intend to simplify an active learning process by integrating a set of evolving learning behaviors into learning models while improving their transparency.

## 2. Results

### 2.1. Design of Experiments

We built a SGD-based CNN classifier with two convolutional layers with a ReLU activation and one dropout layer ending with a softmax layer in PyTorch. The first convolutional layer filters the 1×10 input image with the square kernel of size 5. The second convolutional layer takes as input the pooled output of the first convolutional layer with a stride of 2 pixels and the square kernel of size 5. An SGD optimizer with learning rate 0.01 and momentum 0.5 was trained on $n_{\mathrm{epoch}}=10$ with $n_{\mathrm{batch}}=64$ and tested with $n_{\mathrm{batch}}=1000$.

We tested the CNN model on the MNIST and Fashion MNIST datasets. From each dataset we randomly took $m_{\mathrm{train}}=10,000$ examples for training and $m_{\mathrm{test}}=10,000$ examples for testing. The active learning environment was created with three labeled pool |L|={100,500,1000} with fifty rounds $n_{\mathrm{round}}=50$ and hundred queried examples $|L_{S}|=100$.

The proposed Information Capacity strategy was implemented in line with the two baseline algorithms—Least Confidence and Entropy Sampling. Each experiment was repeated $n_{\mathrm{run}}=10$ in order to produce statistically significant estimates.

### 2.2. Analysis of Experiments

The parameters of the proposed strategy in training the model defined clearly interpretable behavior of learners during multiple-choice testing. The learners (network weights) guessed correctly with the probability $a_{i}=0.1$ on the item (labeled example) *i* of the difficulty $\beta_{i}=4$. We assumed that there was no penalty for guessing announced. The item discrimination parameter $\alpha_{i}=0.25$ reflects how well an item discriminates among the learners located at different points $\theta_{j}$ along the continuum. These values for parameters are chosen to minimize the maximum of the information capacity of the items in *L* but, at the same time, avoid possible inaccuracies caused by machine precision when the informativeness measure values are approaching zero and become imperceptible for different classes.

Implemented guessing behavior reflects “noise” in information. Therefore, a nonzero $a_{i}$ reduced the amount information available for locating learners on the θ continuum. In addition, answering the item *i*, the learners with locations at $\theta_{j}$ did not have a success probability equal to 1 but $b_{i}=0.9$ due to partial forgetting. The locations $\theta_{j}<\beta_{i}$ present lower level learners, while the locations $\theta_{j}\geq \beta_{i}$ describe higher level learners. The given values for the parameters $\alpha_{i},\beta_{i},a_{i},b_{i}$ define the behavior of learners responding to the items in accord with the item information function (see Section 3) that presents the amount of information each item provides.

**Figure 2:**
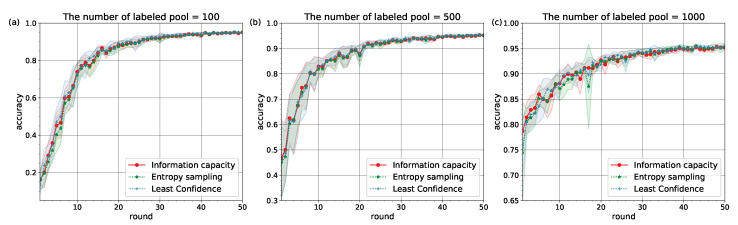
The accuracy curves on MNIST for different numbers of labeled pool: (**a**) |L|=100. (**b**) |L|=500. (**c**) |L|=1000.

**Figure 3:**
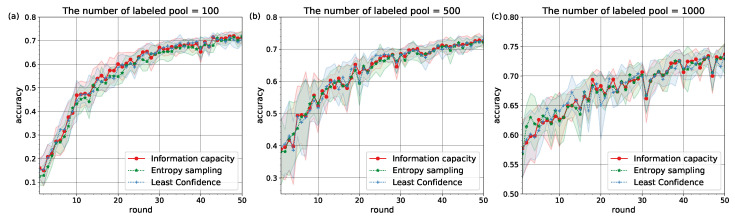
The accuracy curves on Fashion MNIST for different numbers of labeled pool: (**a**) |L|=100. (**b**) |L|=500. (**c**) |L|=1000.

The experiments confirmed that Information Capacity with pre-defined learning behavior can represent the baseline active learning strategies (see Figure 2 and Figure 3). The values of accuracy on testing over rounds mean±std are given in Table 1 and Table 2.

The similarity in learning behavior for different subsets of the MNIST and Fashion MNIST datasets pointed to the conclusion that Information Capacity relies on neural network architectures to model learners’ behavior. It can be explained by the fact that decisions on classification tasks are made at the output layer of a network, but depended on weights (learners) which were set at hidden layers. With increasing amount of labeled pool |L| the similarities between the accuracy curves for different strategies become stronger (see Table 1 and Table 2).

We applied a one-sided Wilcoxon test [44] with Bonferroni correction [45,46] for each round to confirm a lack of statistically significant differences between the three strategies in the accuracy values on testing. Since the *p*-value for each round turned out to be close to 1, we have a sufficient reason to accept the null hypothesis. Consequently, the similarities between the accuracy curves in Figure 2 and Figure 3 are statistically significant.

### 2.3. Discussion

We took Least Confidence and Entropy Sampling for comparison for two reasons. First, these active learning strategies are used as baseline sampling techniques for more complex approaches adopted in deep active learning. Second, Information Capacity shares some similarity with them—it finds $y_{i}$ which range over all possible labels (Entropy Sampling) with the least information capacity (Least Confidence).

As progress on improving performance in deep learning has come at the cost of transparency, we find this approach particularly beneficial. Information Capacity allows learners to exhibit different learning behaviors with regard to the IRT hyperparameters. In the experiments, they were chosen in a certain way to rule out the reasoning behind the Least Confidence and Entropy Sampling strategies. In particular, we modeled uncertainty with a group of learners, who adopted both guessing and forgetting strategies ($a_{i}>0$ and $b_{i}<1$) to classify “hard” items ($\beta_{i}>2$). In addition, it was difficult to assess how strong or weak the learners were ($\theta_{j}<\beta_{i}$ or $\theta_{j}>\beta_{i}$) because the value of discrimination factor was low $\alpha_{i}<0.5$. No penalty for guessing p=0 delivered less predictable behavioral observations.

As we have seen, Least Confidence and Entropy Sampling can be interpreted by the scenario in which each learner (neuron) in a neural network shares the same behavior. Considering the complexity of deep networks, these backbone strategies seem limited. For increasing the transparency of deep learning process, different combinations of the IRT parameters can be used to construct a variety of educational scenarios and learning strategies with strong or weak learners including learning in groups [47,48,49].

The analysis of different neural network architectures with regard to learning behaviors is beyond the scope of this study. However, we hope that our presentation of neural networks will encourage further research exploring novel neural networks building groups of learners with learning behavior which is not limited to gradient-based methods and primitive connectionist models.

## 3. Materials and Methods

### 3.1. Problem Statement

Let X be a feature space and Y be a label space. Let P(X,Y) be an unknown underlying distribution, where X∈X, Y∈Y. We use labeled training set $S_{m}=\left(x_{i},y_{i}\right)$ of *m* labeled training samples to select a prediction function f∈F, f:X→Y so as to minimize the risk $R_{\ell }\left(f\right)=E_{\left(X,Y\right)}\left[\ell \left(f\left(x\right),y\right)\right]$, where $\ell \left(\cdot \right)\in R^{+}$ is a given loss function. For any labeled set *L* (training and testing), the empirical risk over *L* is given by:

$${\hat{r}}_{L}\left(f\right)=\frac{1}{\left|L\right|}\sum_{i=1}^{\left|L\right|}\ell \left(f\left(x_{i}\right),y_{i}\right).$$

In a pool-based setting [7,33], an active learner chooses examples from a set U=m−L of unlabeled samples according to a query function *S*. Query functions often select points based on information inferred from the current model $f_{s}$, the existing training set |L|, and the current pool |U|. The aim is to accurately train the model for a given number of labeled points $|L_{S}|$.

We consider a class B of learning behaviors during testing, where each behavior B∈B represents a hypothesis class containing all learners $f_{s}\in B$, where *s* defines a set of parameters in a testing framework for making behavioral observations.

### 3.2. Testing Framework

We are interested in measuring classification proficiencies of a group of learners (neurons or network weights). Although it seems impossible to directly observe the level of proficiency (working knowledge), we can infer its existence through behavioral observations in a classroom. The learners are given an instrument containing several items (labeled examples) i.e., multiple-choice tests [27,28,50,51,52]. The responses to this instrument constitute the behavioral observations.

Item Response Theory [24,28,53,54,55,56] suggests a variety of models to assess the distance between the learner and the item locations as it clearly defines the learner’s correct response. This means that items located toward the right side have difficulty β. They require a learner to have greater proficiency θ to correctly answer items located on the right side than items located on the left side. In general, items located below 0 are “easy” while items above 0 are “hard”.

In this study, we focused on the four-parameter logistic item response theory (4PL IRT) model which can be presented as [24]:

(1) $$p\left(y_{ij}=1|\theta_{j},\alpha_{i},\beta_{i},a_{i},b_{i}\right)=a_{i}+\frac{b_{i}-a_{i}}{1+\exp(-\alpha_{i}\left(\theta_{j}-\beta_{i}\right))},$$

where $p(y_{ij}=1|\theta_{j},\alpha_{i},\beta_{i},a_{i},b_{i})$ is the probability of providing the correct response $y_{ij}=1$ to an item *i* by a learner *j* with the location (ability) $\theta_{j}$. From the definition (1) we can see that the rate of success mainly depends on the relationship between the item’s parameters and learners’ proficiency.

### 3.3. Information Capacity

So far, we considered the estimation of a learner’s location from its uncertainty. Let us now take the opposite side and define a query strategy *S*.

The instrument’s items—labeled examples—contain a certain amount of information that can be used for estimating the learner location parameters. We assume that each item contributes information to reduce the uncertainty about a learner’s location independent of the other items of the instrument. The amount of information items provide can be presented using the Fisher information as [24,57,58]:

(2) $$S\left(\theta \right)=-E\left[\frac{\partial^{2}}{\partial \theta^{2}}\ln L\right]=\sum_{i=1}^{m}\frac{p_{i}^{\prime 2}}{p_{i}\left(1-p_{i}\right)}=\frac{1}{\sigma_{e}^{2}\left(\hat{\theta }|\theta \right)}$$

where $\sigma_{e}^{2}\left(\hat{\theta }|\theta \right)$ is the asymptotic variance error of the estimate θ. The log likelihood function for a learner *j*’s response vector is equal to:

(3) $$\ln L\left(y_{ij}|\theta_{j},\alpha_{i},\beta_{i},a_{i},b_{i}\right)=\sum_{i=1}^{m}\left(y_{ij}\ln\left(p_{i}\right)+\left(1-y_{ij}\right)\ln\left(1-p_{i}\right)\right),$$

where $p_{i}\equiv p\left(y_{ij}=1|\theta_{j},\alpha_{i},\beta_{i},a_{i},b_{i}\right)$. The items’ capacity is defined as the maximum of the information function $S{\left(\theta \right)}_{\max}$.

The definition (2) can be rewritten with regard to (1) in the explicit form as:

(4) $$S\left(\theta ;\alpha_{i},\beta_{i},a_{i},b_{i}\right)=\sum_{i=1}^{m}\frac{\alpha_{i}^{2}{\left(p_{i}-a_{i}\right)}^{2}{\left(\left(1-p_{i}\right)\left(b_{i}-a_{i}\right)-\left(p_{i}-a_{i}\right)\left(1-b_{i}\right)\right)}^{2}}{p_{i}\left(1-p_{i}\right){\left(b_{i}-a_{i}\right)}^{2}{\left(1-a_{i}\right)}^{2}}.$$

**Figure 4:**
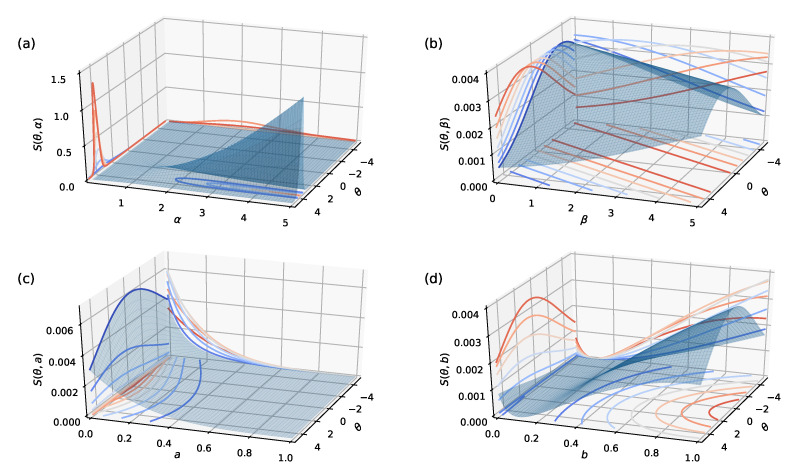
The projections of the information function on different planes: (**a**) α-plane with α=[0,5]. (**b**) β-plane with β=[0,5]. (**c**) *a*-plane with a=[0,1]. (**d**) *b*-plane with b=[0,1].

The detailed derivation of the Equation (4) is given in Appendix A. Figure 4 illustrates the projections of the information function with fixed values α=0.25, β=4, a=0.1, b=0.9 described in Section 2.2.

**Figure 5:**
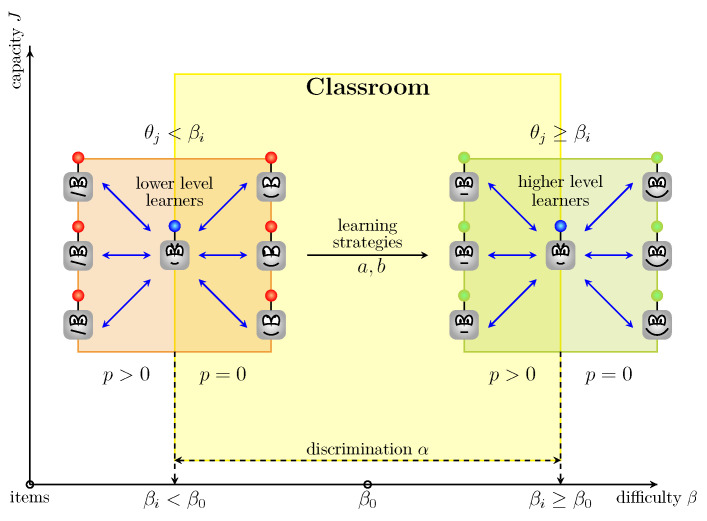
The proposed “classroom” strategy.

A new pool-based strategy—we refer to as Information Capacity—suggests estimating the information capacity for unlabelled instances based on the definition (4). Figure 5 depicts the proposed pool-based strategy for measuring the items capacity *S* with regard to the items’ difficulty $\beta_{i}$, learners’ locations $\theta_{j}$, strategies $a_{i}$ and $b_{i}$, and penalty announcement *p* in a classroom. The strategy is aimed at “moving” learners along the difficulty axis while keeping high values for the capacity axis. The learners query the examples with the lowest information capacity. For clarity, the algorithm represents the proposed pool-based active learning with the Information Capacity strategy (see Algorithm 1). The differences in implementation in comparison with the traditional active learning framework are highlighted in blue.
**Algorithm 1** Pool-based active learning with Information Capacity strategy1:**procedure**InformationCapacity($m_{\mathrm{train}}$, $m_{\mathrm{test}}$)    2:    Initialize a labeled training set *L*;  3:    Initialize an unlabeled training pool $U=m_{\mathrm{train}}-L$;  4:    Initialize a learning behavior of learners *B* with regard to a set of parameters α,β,a,b;  5:    Train a group of learners on the labeled set *L*;  6:    Measure performance of the group of learners on the test set $m_{\mathrm{test}}$;  7:    Initialize several rounds $n_{\mathrm{round}}$ and several queried examples $|L_{S}|$;   8:    **for** $round\in n_{\mathrm{round}}$ **do** 9:        Estimate the probabilities with regard to (1) based on the learning behavior *B*;  10:        Sort the unlabeled items in *U* according to (4) based on the probabilities from the step 9;  11:        Query the items $L_{S}$ with the smallest of the maximum capacity *S* in a round;  12:        $L\leftarrow L\cup L_{S}$;  13:        $U\leftarrow U\backslash L_{S}$;  14:        Retrain a group of learners on the labeled set *L*;  15:        Measure performance of the group of learners on the test set $m_{\mathrm{test}}$;  16:    **end for**  17:    **return** The performance of the learners with the interpretation of their learning behavior.  

## 4. Conclusions

We present Information Capacity, which is an uncertainty sampling strategy that effectively integrates human- and machine-reasoning processes. The strategy allows embedding into the models different learning behaviors with regard to the parameters of the 4PL IRT model. The experiments on the MNIST and Fashion MNIST datasets with the same CNN model indicate that Information Capacity performs similarly to Least Confidence and Entropy Sampling but brings more transparency into a deep learning process.

We considered the neurons or network weights of the CNN classifier at the last hidden layer as a group of learners with different proficiency in classifying learning items, i.e., images. The pre-defined parameters of the Information Capacity strategy defined their learning behavior: the learners had a success probability $b_{i}=0.9$ due to partial forgetting while they guessed correctly with the probability $a_{i}=0.1$ on the item *i* of the difficulty $\beta_{i}=4$, which discriminated the learners with the factor $\alpha_{i}=0.25$.

The equivalence of the parameters $\alpha_{i},\beta_{i},a_{i},b_{i}$ for different subsets of the MNIST and Fashion MNIST datasets revealed that the model architecture greatly influences learning behavior. As a direction for further research, we suggest modeling learning behaviors with different network architectures. While keeping equally good performance due to the similarity between different strategies, it seems desirable to optimize neural network architectures and learning processes.

The code used for empirical evaluation is available at https://github.com/yukinoi/human-like-active-learning.

## Figures and Tables

**Table 1:** The values of accuracy on testing over rounds mean±std on MNIST.

Strategy	|L|=100	|L|=500	|L|=1000
Information Capacity	0.8135±0.0254	0.8714±0.0279	0.9153±0.0117
Entropy Sampling	0.8071±0.0259	0.8688±0.0264	0.9133±0.0141
Least Confidence	0.8182±0.0276	0.8727±0.0275	0.9156±0.0126

**Table 2:** The values of accuracy on testing over rounds mean±std on Fashion MNIST.

Strategy	|L|=100	|L|=500	|L|=1000
Information Capacity	0.5646±0.0399	0.6279±0.0397	0.6779±0.0245
Entropy Sampling	0.5499±0.0362	0.6252±0.0401	0.678±0.0254
Least Confidence	0.5615±0.0387	0.6273±0.04	0.677±0.0255

## Appendix A. Derivation of the Equation (4)

Let us denote $p_{i}\equiv p\left(y_{ij}=1|\theta_{j},\alpha_{i},\beta_{i},a_{i},b_{i}\right)$. The derivative of the model (1) can be defined as

(A1) $$p_{i}^{\prime }=\frac{\alpha_{i}\left(b_{i}-a_{i}\right)\exp\left(-\alpha_{i}\left(\theta_{j}-\beta_{i}\right)\right)}{{\left(1+\exp(-\alpha_{i}\left(\theta_{j}-\beta_{i}\right))\right)}^{2}}.$$

Extracting the definition (1) from (A1) gives

(A2) $$p_{i}^{\prime }=\alpha_{i}\left(p_{i}-a_{i}\right)\frac{\exp(-\alpha_{i}\left(\theta_{j}-\beta_{i}\right))}{1+\exp(-\alpha_{i}\left(\theta_{j}-\beta_{i}\right))}.$$

Let us now define the probability of getting an incorrect response:

(A3) $$1-p_{i}=\frac{\left(1-a_{i}\right)\exp\left(-\alpha_{i}\left(\theta_{j}-\beta_{i}\right)\right)}{1+\exp(-\alpha_{i}\left(\theta_{j}-\beta_{i}\right))}-\frac{1-b_{i}}{1+\exp(-\alpha_{i}\left(\theta_{j}-\beta_{i}\right))}.$$

From the Equation (A3), it follows that

(A4) $$\frac{\exp(-\alpha_{i}\left(\theta_{j}-\beta_{i}\right))}{1+\exp(-\alpha_{i}\left(\theta_{j}-\beta_{i}\right))}=\frac{\left(1-p_{i}\right)\left(b_{i}-a_{i}\right)-\left(p_{i}-a_{i}\right)\left(1-b_{i}\right)}{\left(b_{i}-a_{i}\right)\left(1-a_{i}\right)}.$$

Taking into account (A4), we can rewrite (A1) as follows:

(A5) $$p_{i}^{\prime }=\alpha_{i}\left(b_{i}-a_{i}\right)\frac{\left(1-p_{i}\right)\left(b_{i}-a_{i}\right)-\left(p_{i}-a_{i}\right)\left(1-b_{i}\right)}{\left(b_{i}-a_{i}\right)\left(1-a_{i}\right)}.$$

Substituting (A5) into the definition (2) immediately gives (4).

## References

[1] Bachman P., Sordoni A., Trischler A. Learning algorithms for active learning. Proceedings of the 34th International Conference on Machine Learning.

[2] Fazakis N., Kanas V.G., Aridas C.K., Karlos S., Kotsiantis S. (2019). Combination of active learning and semi-supervised learning under a self-training scheme. Entropy.

[3] Hsu W.-N., Lin H.-T. Active learning by learning. Proceedings of the Twenty-Ninth AAAI Conference on Artificial Intelligence.

[4] Huang S., Jin R., Zhou Z. (2014). Active learning by querying informative and representative examples. IEEE Trans. Pattern Anal. Mach. Intell..

[5] Konyushkova K., Raphael S., Fua P. Learning active learning from data. Proceedings of the Conference on Neural Information Processing Systems (NIPS).

[6] Ramirez-Loaiza M.E., Sharma M., Kumar G., Bilgic M. (2017). Active learning: An empirical study of common baselines. Data Min. Knowl. Discov..

[7] Settles B. (2009). Active Learning Literature Survey. Computer Sciences Technical Report.

[8] Sourati J., Akcakaya M., Dy J.G., Leen T.K., Erdogmus D. (2016). Classification Active Learning Based on Mutual Information. Entropy.

[9] Sourati J., Akcakaya M., Leen T.K., Erdogmus D., Dy J.G. (2017). A probabilistic active learning algorithm based on Fisher information ratio. IEEE Trans. Pattern Anal. Mach. Intell..

[10] Wu J., Sheng V.S., Zhang J., Li H., Dadakova T., Swisher C.L., Cui Z., Zhao P. (2020). Multi-label active learning algorithms for image classification: Overview and future promise. ACM Comput. Surv..

[11] Joshi A.J., Porikli F., Papanikolopoulos N. Multi-class active learning for image classification. Proceedings of the Conference on Computer Vision and Pattern Recognition.

[12] Yang Y., Ma Z., Nie F., Chang X., Hauptmann A.G. (2015). Multi-Class active learning by uncertainty sampling with diversity maximization. Int. J. Comput. Vis..

[13] Hanneke S. (2012). Activized Learning: Transforming passive to active with improved label complexity. J. Mach. Learn. Res..

[14] Bonwell C., Eison J. (1991). Active Learning: Creating Excitement in the Classroom.

[15] Cook B.R., Babon A. (2017). Active learning through online quizzes: Better learning and less (busy) work. J. Geogr. High. Educ..

[16] Prince M. (2004). Does Active Learning Work? A review of the research. J. Eng. Educ..

[17] Aubrey K., Riley A. (2015). Understanding and Using Educational Theories.

[18] Mascharka D., Tran P., Soklaski R., Majumdar A. Transparency by design: Closing the gap between performance and interpretability in visual reasoning. Proceedings of the 2018 IEEE/CVF Conference on Computer Vision and Pattern Recognition (CVPR).

[19] Castro R., Kalish C., Nowak R., Qian R., Rogers T., Zhu X. Human active learning. Proceedings of the Twenty-Second Annual Conference on Neural Information Processing Systems.

[20] Lake B.M., Salakhutdinov R., Tenenbaum J.B. (2015). Human-level concept learning through probabilistic program induction. Science.

[21] Lake B.M., Ullman T.D., Tenenbaum J.B., Gershman S.J. (2017). Building machines that learn and think like people. Behav. Brain Sci..

[22] Mastorakis G. (2018). Human-like machine learning: Limitations and suggestions. arXiv.

[23] Wilson R.C., Shenhav A., Straccia M., Cohen J.D. (2019). The Eighty Five Percent Rule for optimal learning. Nat. Commun..

[24] De Ayala R.J. (2009). The Theory and Practice of Item Response Theory (Methodology in the Social Sciences).

[25] Gierl M.J., Bulut O., Guo Q., Zhang X. (2017). Developing, analyzing, and using distractors for multiple-choice tests in education: A comprehensive review. Rev. Educ. Res..

[26] Hakel M.D. (1998). Beyond Multiple Choice: Evaluating Alternatives to Traditional Testing for Selection.

[27] Lee C.J. (2019). The test taker’s fallacy: How students guess answers on multiple-choice tests. Behav. Decis. Mak..

[28] Lord F.M. (1980). Applications of Item Response Theory to Practical Testing Problems.

[29] Thissen D., Steinberg L., Fitzpatrick A.R. (1989). Multiple-choice models: The distractors are also part of the item. J. Educ. Meas..

[30] Mittelstadt B., Russell C., Wachter S. Explaining explanations in AI. Proceedings of the Fairness, Accountability, and Transparency (FAT*).

[31] Rudin C. (2019). Stop explaining black box machine learning models for high stakes decisions and use interpretable models instead. Nat. Mach. Intell..

[32] Gal Y., Islam R., Ghahramani Z. Deep Bayesian active learning with image data. Proceedings of the 34th International Conference on MachineLearning.

[33] Geifman Y., El-Yaniv R. Deep active learning with a neural architecture search. Proceedings of the 33rd Conference on Neural Information Processing Systems (NeurIPS 2019).

[34] Ouali Y., Hudelot C., Tami M. (2020). An overview of deep semi-supervised learning. arXiv.

[35] Sener O., Savarese S. Active learning for convolutional neural networks: A core-set approach. Proceedings of the 6th International Conference on Learning Representations (ICLR 2018).

[36] Wang D., Shang Y. A new active labeling method for deep learning. Proceedings of the International Joint Conference on Neural Networks (IJCNN).

[37] Budd S., Robinson E.C., Kainz B. (2019). A survey on active learning and human-in-the-loop deep learning for medical image analysis. arXiv.

[38] Chen Y., Filho T.S., Prudencio R.B.C., Diethe T., Flach P. (2019). *β*^3^-IRT: A new item response model and its applications. arXiv.

[39] Martinez-Plumed F., Prudencio R.B.C., Martinez-Uso A., Hernandez-Orallo J. (2019). Item response theory in AI: Analysing machine learning classifiers at the instance Level. Artif. Intell..

[40] Whitehill J., Ruvolo P., Wu T., Bergsma J., Movellan J. Whose vote should count more: Optimal integration of labels from labelers of unknown expertise. Proceedings of the Advances in Neural Information Processing Systems 22 (NIPS 2009).

[41] Yeung C.K. (2019). Deep-IRT: Make deep learning based knowledge tracing explainable using item response theory. arXiv.

[42] Lalor J.P., Wu H., Yu H. (2017). CIFT: Crowd-informed fine-tuning to improve machine learning ability. arXiv.

[43] Ravi S., Larochelle H. (2018). Meta-Learning for Batch Mode Active Learning. https://openreview.net/forum?id=r1PsGFJPz.

[44] Wilcoxon F. (1945). Individual comparisons by ranking methods. Biom. Bull..

[45] Dunn O.J. (1961). Multiple comparisons among means. J. Am. Stat. Assoc..

[46] Perneger T.V. (1998). What’s wrong with Bonferroni adjustments. BMJ.

[47] Kulikovskikh I.M., Prokhorov S.A., Suchkova S.A. (2017). Promoting collaborative learning through regulation of guessing in clickers. Comput. Hum. Behav..

[48] Le H., Janssen J., Wubbels T. (2018). Collaborative learning practices: Teacher and student perceived obstacles to effective student collaboration. Camb. J. Educ..

[49] Sawyer J., Obeid R., Obeid R., Schwartz A., Shane-Simpson C., Brooks P.J. (2018). Cooperative and collaborative learning: Getting the best of both words. How We Teach Now: The GSTA Guide to Student-Centered Teaching.

[50] Liu C.W., Wang W.C. (2016). Unfolding IRT models for Likert-type items with a don’t know option. Appl. Psychol. Meas..

[51] Liu C.W., Wang W.C. (2019). A general unfolding IRT model for multiple response styles. Appl. Psychol. Meas..

[52] Sideridis G., Tsaousis I., Harbi K.A. (2016). Improving measures via examining the behavior of distractors in multiple-choice tests: Assessment and remediation. Educ. Psychol. Meas..

[53] Bonifay W. (2020). Multidimensional Item Response Theory (Quantitative Applications in the Social Sciences).

[54] DeMars C.E. (2007). “Guessing” parameter estimates for multidimensional Item Response Theory models. Educ. Psychol. Meas..

[55] Gin B., Sim N., Skrondal A., Rabe-Hesketh S. (2019). A dyadic IRT model. arXiv.

[56] Reckase M.D. (2009). Multidimensional Item Response Theory.

[57] Frieden B.R. (2004). Science from Fisher Information: A Unification.

[58] Lehmann E.L., Casella G. (1998). Theory of Point Estimation.

